# Olfactory Radioanatomical Findings in Patients With Cardiac Arrhythmias, COVID-19, and Healthy Controls

**DOI:** 10.7759/cureus.26564

**Published:** 2022-07-05

**Authors:** Mitchell R Gore

**Affiliations:** 1 Otolaryngology - Head and Neck Surgery, State University of New York Upstate Medical University, Syracuse, USA

**Keywords:** sinus disease, olfactory, olfactory fossa, olfactory dysfunction, olfactory bulb

## Abstract

Background

Clinical hyposmia and anosmia are commonly seen, most frequently with either post-inflammatory, age-related, or idiopathic causes being most frequent. Actual anatomical abnormalities of the olfactory groove or olfactory bulb are far less common. A recent case report showing a possible link between congenital olfactory bulb agenesis and Wolff-Parkinson-White syndrome suggested that there may be a relationship between cardiac arrhythmia and olfactory bulb development. While Kallmann syndrome (KS) is the classic syndrome involving olfactory bulb agenesis and hypogonadotropic hypogonadism, this case report and a prior report noting isolated hypogonadotropic hypogonadism and the Wolff-Parkinson-White syndrome suggest there may be more rare associations between cardiac arrhythmia and olfactory groove abnormalities.

Methods

A retrospective study was conducted to attempt to elucidate whether there may be a link between cardiac arrhythmias and olfactory anatomical abnormalities. The olfactory bulb volume (OBV) and olfactory sulcus depth (OSD) of 44 patients with cardiac arrhythmias were compared to 43 healthy control patients. Additionally, 11 patients with acute COVID-19 were also compared in those groups. Patients were seen between September and December 2020. Available MRI images were utilized.

Results

The average right and left olfactory bulb volume was 29.42±18.17 mm^3^ and 25.67±15.29 mm^3^ for patients with cardiac arrhythmia, 40.79±30.65 mm^3^ and 38.95±21.87mm^3^ for healthy controls, and 21.30±15.23 mm^3^ and 17.75±9.63 mm^3^ for COVID-19 patients. The average right and left olfactory sulcus depth was 7.68±1.31 mm and 7.47±1.56 mm for patients with cardiac arrhythmia, 10.67±1.53 mm and 10.62±1.67 mm for controls, and 7.91±0.99 mm and 8.02±0.88 mm for COVID-19 patients. The right and left olfactory bulb volume difference versus controls was significant for cardiac arrhythmia patients (p=0.028 and p=0.0038) and for COVID-19 patients (p=0.047 and p=0.0029), and the right and left olfactory sulcus depth difference versus controls was significant for cardiac arrhythmia patients (p<0.0001 and p<0.0001) and for COVID-19 patients (p<0.0001 and p<0.0001). Both COVID-19 and cardiac arrhythmia patients were, on average, significantly older than controls. On multivariate analysis, cardiac arrhythmia or COVID-19 diagnosis did not significantly correlate with smaller olfactory bulb volume, but older age, cardiac arrhythmia diagnosis, and COVID-19 diagnosis did significantly correlate with smaller olfactory sulcus depth. On multivariate analysis, older age was significantly correlated with cardiac arrhythmia diagnosis and COVID-19 diagnosis.

Conclusions

Olfactory bulb volume and olfactory sulcus depth in both cardiac arrhythmia and COVID-19 patients appeared significantly smaller than in controls. Cardiac arrhythmia and COVID-19 patients were significantly older than controls. Age, as well as genetic predisposition, may contribute to a difference in the radiographic olfactory anatomical findings in patients with cardiac arrhythmias and COVID-19.

## Introduction

A recent case report [1] noted an adult patient with previously undiagnosed congenital anosmia as well as the radiographic absence of the olfactory groove/bulbs as well as Wolff-Parkinson-White syndrome. Further investigation revealed a prior case report [2] involving a patient with isolated hypogonadotropic hypogonadism, pronounced hypodontia, and the Wolff-Parkinson-White syndrome. The classic Kallmann syndrome (KS) involves hypogonadotropic hypogonadism and olfactory bulb aplasia. The presence of one of the two classic signs of Kallmann syndrome in the aforementioned case reports but not both, while both involved Wolff-Parkinson-White syndrome, prompted an investigation into whether there may be an association between cardiac arrhythmia in general and olfactory nerve abnormalities [3-7]. The gonadotropin‐releasing hormone‐1 (GnRH) system is involved in the development of both the reproductive and olfactory systems, which may contribute to the concomitant reproductive and olfactory dysfunction seen in Kallmann syndrome patients [4,5]. Human cardiac tissue and cardiac-associated immune cells have been shown to contain GnRH receptors, and studies in cephalopods have suggested that GnRH may have receptor targets in the cardiovascular system, which may explain the possible link between cardiac arrhythmias and olfactory nerve abnormalities. Additionally, a recent study [8] on MRI and CT findings in patients with COVID-19-related anosmia noted that radiographic olfactory changes included olfactory cleft opacification, decreased olfactory bulb volumes (OBVs), and olfactory bulb signal abnormalities such as increased signal intensity, hyperintense foci, and microhemorrhages. Olfactory bulb volume and olfactory sulcus depth (OSD) have been shown to be altered in myriad conditions, from septo-optic dysplasia to depression, post-infectious anosmia/hyposmia, and many others [9-17]. This retrospective study aimed to determine whether patients with cardiac arrhythmias and patients with acute COVID-19 had decreased olfactory bulb volume and olfactory sulcus depth relative to healthy controls.

## Materials and methods

**Figure 1:**
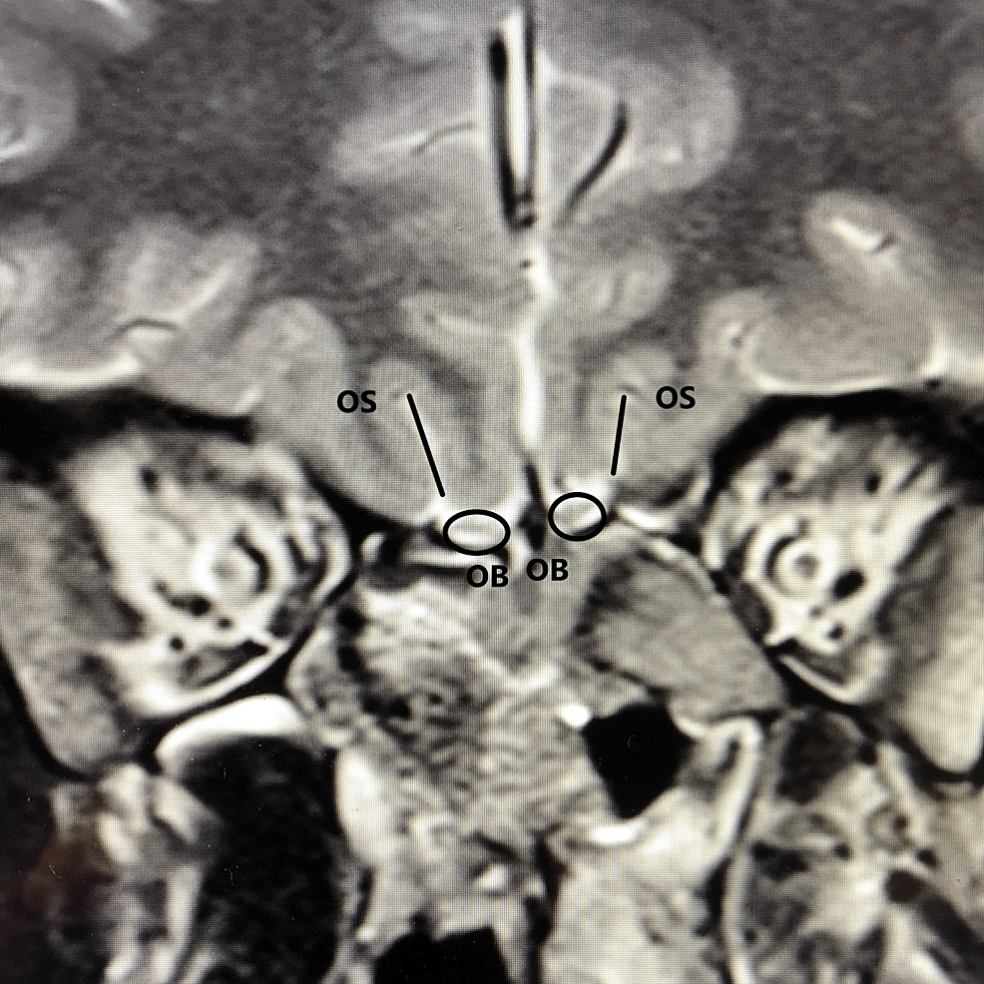
Coronal T2 MRI showing the olfactory bulb and olfactory sulcus.

The patient data were collected through a retrospective review of the records of patients who presented to a university hospital between September 2020 and December 2020, underwent head/brain MRI, and fit the study inclusion and exclusion criteria. Between September and December 2020, the head/brain or maxillofacial MRI of 44 patients with cardiac arrhythmias, 43 healthy control patients, and 11 patients with acute COVID-19 were analyzed. Patients aged 18 years or older were included in the three groups. Cardiac arrhythmia patients were analyzed if they had a current diagnosis of any cardiac arrhythmia and had an available head/brain or maxillofacial MRI completed between September and December 2020. COVID-19 patients were analyzed if they had a current diagnosis of acute COVID-19 and had an available head/brain or maxillofacial MRI completed between September and December 2020. Healthy control patients were analyzed if they had an available head/brain or maxillofacial MRI completed between September and December 2020 and did not carry a current diagnosis of any cardiac arrhythmia, COVID-19, disorders of smell/taste, anosmia, hyposmia, or head trauma. Patient medications were analyzed to exclude patients taking medications that could cause anosmia/hyposmia such as intranasal zinc medications, topical decongestant intranasal sprays, and oral medications such as phenothiazines or nifedipine. Figure 1 shows a coronal MRI image illustrating the olfactory bulb and the olfactory sulcus. OBVs were calculated using volumetric analysis of the olfactory bulb on T2 MRI sequences as previously described [12] using the 3D Slicer software ver. 4.10.2 (http://www.slicer.org/). The 3D slicer software is a free, open-source software package for the analysis of medical imaging developed by Harvard University and facilitated volumetric analysis of the olfactory bulb data. The olfactory bulbs were segmented by tracing their outlines manually, and the software ran a quantification process that rendered the volume of the olfactory bulb. OSD was measured as described previously [8] on coronal T2 images by measuring the depth to the deepest point of the olfactory sulcus along a line tangent to the inferior borders of the gyrus rectus. In addition to patient diagnosis and olfactory bulb volume and sulcus depth, data on patient age and gender were compared. Patient data were de-identified and retrospective, and this study was approved by the SUNY-Upstate Institutional Review Board (1427574-1).

Statistical analyses

Patient data were compiled in Microsoft Excel (Microsoft Corporation, Redmond, Washington, USA) and the data were analyzed using XLSTAT (Addinsoft, Paris, France). Continuous variables were analyzed using the Student’s t-test and one-way analysis of variance (ANOVA) for comparison between groups. The Pearson Correlation/Association test was also utilized to determine the correlation between the observed data variables. Multivariate analysis was conducted via logistical regression using XLSTAT, utilizing a Newton-Raphson algorithm. The level of statistical significance was set at p < 0.05.

## Results

Table 1 shows the patient characteristics for each group. Of the 44 cardiac arrhythmia patients, 38 had atrial fibrillation only, one had atrial fibrillation and supraventricular tachycardia, three had atrial flutter, one had sick sinus syndrome, and one had prolonged Q-T syndrome. Table 2 shows the olfactory bulb volume and olfactory sulcus depth, patient age, and patient sex data and univariate analysis data for the three patient groups. The average right and left olfactory bulb volume was 29.42±18.17 mm^3^ and 25.67±15.29 mm^3^ for patients with cardiac arrhythmia, 40.79±30.65 mm^3^ and 38.95±21.87mm^3^ for healthy controls, and 21.30±15.23 mm^3^ and 17.75±9.63 mm^3^ for COVID-19 patients. The average right and left olfactory sulcus depth was 7.68±1.31 mm and 7.47±1.56 mm for patients with cardiac arrhythmia, 10.67±1.53 mm and 10.62±1.67 mm for healthy controls, and 7.91±0.99 mm and 8.02±0.88 mm for COVID-19 patients. The right and left olfactory bulb volume difference versus controls was significant for cardiac arrhythmia patients (p=0.028 and p=0.0038) and for COVID-19 patients (p=0.047 and p=0.0029), and the right and left olfactory sulcus depth difference versus controls was significant for cardiac arrhythmia patients (p<0.0001 and p<0.0001) and for COVID-19 patients (p<0.0001 and p<0.0001). Multivariate analysis via XLSTAT utilizing logistical regression of the data using an iterative algorithm using the Newton-Raphson algorithm was performed. The multivariate analysis data are shown in Table 3. On multivariate analysis, age (p=0.001) and cardiac arrhythmia diagnosis (p=0.0001) or COVID-19 diagnosis (p=0.0001) remained significant predictors of smaller olfactory sulcus depth but not of smaller olfactory bulb volume. Patient sex was not a significant predictor of olfactory sulcus depth or olfactory bulb volume on multivariate analysis. The average age for the cardiac arrhythmia group was 76.11±13.13 years (p<0.0001 vs control group), 51.86±17.66 years for the control group, and 69.27±17.64 years for the COVID-19 group (p=0.0005 vs. control group). Of the 44 cardiac arrhythmia patients, 28 were male and 16 were female. Of the 43 control patients, 21 were male and 22 were female. Of the 11 COVID-19 patients, six were male and five were female.

**Table 1:** Patient characteristics for the study patients.

Patient characteristics	n
Cardiac arrhythmia (total)	44
Atrial fibrillation	38
Atrial fibrillation + supraventricular tachycardia	1
Atrial flutter	3
Sick sinus syndrome	1
Prolonged Q-T syndrome	1
Controls (total)	43
Covid-19 (total)	11

**Table 2:** Olfactory bulb volume and olfactory sulcus depth data for the cardiac arrhythmia, control, and COVID-19 patients.

Cardiac arrhythmia		
Average right olfactory bulb volume	29.42±18.17 mm^3^	p=0.028 vs. control
Average left olfactory bulb volume	25.67±15.29 mm^3^	p=0.0038 vs. control
Average right olfactory sulcus depth	7.68±1.31 mm	p<0.0001 vs. control
Average left olfactory sulcus depth	7.47±1.56 mm	p<0.0001 vs. control
Average age	76.11±13.13	p<0.0001 vs. control
Male/female	28:16	
Controls		
Average right olfactory bulb volume	40.79±30.65 mm^3^	
Average left olfactory bulb volume	38.95±21.87mm^3^	
Average right olfactory sulcus depth	10.67±1.53 mm	
Average left olfactory sulcus depth	10.62±1.67 mm	
Average age	51.86±17.66	
Male/female	21:22	
COVID-19		
Average right olfactory bulb volume	21.30±15.23 mm^3^	p=0.047 vs. control
Average left olfactory bulb volume	17.75±9.63 mm^3^	p=0.0029 vs. control
Average right olfactory sulcus depth	7.91±0.99 mm	p<0.0001 vs. control
Average left olfactory sulcus depth	8.02±0.88 mm	p<0.0001 vs. control
Average age	69.27±17.64	p=0.0005 vs. control
Male/female	6:5	

**Table 3:** Multivariate analysis of olfactory sulcus depth and olfactory bulb volume data. OSD: olfactory sulcus depth, OBV: olfactory bulb volume.

Multivariate analysis	Variable p-value		Cardiac arrhythmia diagnosis	COVID-19 diagnosis
	Age	Sex		
Left OSD	p=0.001	p=0.5	p=0.0001	p=0.0001
Right OSD	p=0.001	p=0.4	p=0.0001	p=0.0001
Left OBV	p=0.2	p=0.4	p=0.3	p=0.2
Right OBV	p=0.1	p=0.2	p=0.5	p=0.6

## Discussion

The volume of the olfactory bulbs and the depth of the olfactory sulcus are readily obtained from MRI imaging and can be used as a neuroanatomical comparative tool to assess the structure of the olfactory system in patients [18,19]. Olfactory bulb volumes and olfactory sulcus depth values [8,20] vary by patient population, MRI protocol, and measurement/calculation method but are typically on the order of 30-90 mm3 for olfactory bulb volumes and 5-10 mm for olfactory sulcus depth, similar to the average values noted in the patient population in this study. Isolated olfactory nerve agenesis is rare, as in a case report in a 12-year-old girl by Carswell et al. [21], noting a patient with congenital complete absence of the olfactory nerves. Coimbra et al. [3] also reported a similarly rare case of isolated olfactory bulb agenesis. The human olfactory apparatus develops during the fetal stage, and the developing fetus can detect odors as early as 28 weeks, and the developing olfactory bulbs can be seen on MRI at this point. Olfactory axons project from the nasal epithelium prior to the formation of the olfactory bulbs and lack a peripheral ganglion, but the synaptic structures of the future olfactory bulb have this functionality. The olfactory bulb begins to laminate at 14 weeks, but complete myelination occurs postnatally. The olfactory system does not contain direct thalamic projections, but the olfactory bulb and anterior olfactory nucleus essentially serve as thalamic surrogates. Olfactory abnormalities can be seen in children with brain malformations, endocrine disorders, chromosome anomalies, and craniofacial abnormalities [4-6]. Kallmann syndrome is a classically described syndrome presenting with congenital olfactory bulb agenesis. Kallmann syndrome is a subtype of the broader group of isolated gonadotropin-releasing hormone (GnRH) deficiency (IGD) syndromes [7]. KS consists of hypogonadotropic hypogonadism with anosmia and a congenital absence of the olfactory bulbs. There are also less severe and somewhat more common pathologies seen in IGD, including hypothalamic amenorrhea (HA), constitutional delay of puberty (CDP), and adult-onset hypogonadotropic hypogonadism (AHH). The association between hypothalamic hypogonadism and olfactory bulb agenesis in Kallmann syndrome is thought to be related to the association between the GnRH neurons and the olfactory placode. IGD can also be related to non-reproductive features such as midline facial defects, renal agenesis, limb abnormalities, hearing loss, and eye movement and balance disorders.

Acquired olfactory dysfunction can be commonly seen in post-upper respiratory infection (URI) anosmia or hyposmia [10]. Studies have shown that olfactory bulb volume and olfactory sulcus depth decreased in patients with olfactory loss after URI compared to normal controls. Studies have also shown that there may be significant gray matter volume loss in the right orbitofrontal cortex (OFC) in patients with post-infectious olfactory disfunction and that there may be a significant negative correlation between the volume of gray matter in the right OFC as well as olfactory bulb volume with the duration of olfactory loss in these post-infectious olfactory loss patients versus normal controls. Kandemirli et al. [8] examined olfactory function and CT and MRI findings in patients with persistent COVID-19 olfactory dysfunction. They evaluated olfactory function with the Sniffin' Sticks test and collected quantitative measurements of olfactory bulb volumes, olfactory sulcus depths, and olfactory radiographic characteristics. They noted frequent olfactory cleft opacification (~73.9% of cases), subnormal olfactory bulb volumes in ~43.5% of cases, and shallow olfactory sulci in ~60.9% of cases. They also noted frequent abnormalities in olfactory bulb shape, olfactory bulb signal intensity, and frequent microhemorrhages and abnormalities in the clumping of or scarcity of olfactory filia. Studies have also shown that olfactory bulb volume can be decreased in patients with depression, after transsphenoidal pituitary surgery, in patients with Parkinson’s disease, and that olfactory bulb volume can be decreased in women and with increasing age [11-17].

In this study, olfactory bulb volume and olfactory sulcus depth in patients with cardiac arrhythmia, acute COVID-19, and healthy controls were measured. Patients with cardiac arrhythmia and COVID-19 had significantly smaller right and left olfactory bulb volumes and olfactory sulcus depths than controls on univariate analysis and were significantly older than controls. On multivariate analysis, olfactory bulb volume did not correlate significantly with cardiac arrhythmia diagnosis or COVID-19 diagnosis. On multivariate analysis, smaller right and left olfactory sulcus depth did significantly correlate with cardiac arrhythmia and COVID-19 diagnosis. On multivariate analysis, older age was also significantly correlated with cardiac arrhythmia and COVID-19 diagnosis. This may indicate that there may be a correlation between the propensity to develop cardiac arrhythmia and the propensity for olfactory dysfunction or atrophy of the olfactory bulb and/or olfactory sulcus over time. This study's limitations include its retrospective nature, which introduces the possibility of recall and selection bias. Given the retrospective nature of this study, there was some heterogeneity in the MRI studies/sequences available for patients in this study. A prospective study in which all patients had a uniform fine-cut MRI protocol standardized for the study protocol and specifically targeted at the olfactory anatomy would be helpful. Additionally, the relatively low patient numbers are a limitation and may limit power in the statistical analysis. Patient medication lists were screened to exclude patients on intranasal or oral medications that may affect olfaction, but the use of medications not reported by patients and not present in the medical record or use of other patient medications that might unknowingly affect olfaction is another possible limitation. The mild diversity in the arrhythmia types in the cardiac arrhythmia group (although the vast majority were atrial fibrillation patients) and the mild heterogeneity in the diagnoses of the control group may also limit the statistical analysis. Future prospective studies with larger patient numbers and a greater diversity of other cardiac arrhythmia types with distinct statistical analyses for each arrhythmia type (e.g., a large number of purely Wolff-Parkinson-White patients) would be of use. The significantly older age of the cardiac arrhythmia and COVID-19 patients may also act as a confounder, and indeed, on multivariate analysis, older age did significantly correlate with smaller olfactory sulcus depth, as did cardiac arrhythmia diagnosis and COVID-19 diagnosis. This may indicate that cardiac arrhythmia, COVID-19 diagnosis or susceptibility, and older age may all correlate significantly with small olfactory sulcus depth, and that older age is also independently correlated with a propensity for cardiac arrhythmia. Additionally, the collection and availability of a formal, standardized olfactory function measurement in all patients, such as Sniffin’ sticks or the Pittsburgh Smell Identification test, would also allow useful correlation between functional/clinical olfactory data (quantitative olfactory measurements) and radiographic olfactory bulb volume and olfactory sulcus depth data.

## Conclusions

This retrospective radiographic study demonstrated smaller olfactory bulb volumes and olfactory sulcus depths on MRI in patients with a history of cardiac arrhythmia and patients with COVID-19 compared to healthy control patients. Cardiac arrhythmia and COVID-19 patients were significantly older than controls. Multivariate analysis demonstrated that cardiac arrhythmia diagnosis and COVID-19 diagnosis, as well as older age, were all significantly associated with smaller olfactory sulcus depth but not with smaller olfactory bulb volume. Future prospective studies with standardized MRI protocols and larger groups of patients with cardiac arrhythmias and larger numbers of healthy controls may help elucidate whether there is a correlation between a predisposition to cardiac arrhythmia and radiographic abnormalities in the olfactory bulb/olfactory sulcus.
